# Towards attack tolerant networks: Concurrent multipath routing and the butterfly network

**DOI:** 10.1371/journal.pone.0214292

**Published:** 2019-04-03

**Authors:** Edward L. Platt, Daniel M. Romero

**Affiliations:** School of Information, University of Michigan, Ann Arbor, Michigan, United States of America; Rutgers The State University of New Jersey, UNITED STATES

## Abstract

It is crucial for large-scale communication networks such as the internet to be resilient against attacks such as censorship and surveillance, which pose a threat to free expression and free association. Self-organized networks such as the internet’s router network typically have heavy-tailed degree distributions, making them highly vulnerable to targeted attacks against central nodes. While cryptographic solutions exist, they fail to address the underlying topological problem, and remain vulnerable to man-in-the-middle attacks and coercion. Coercion-resistant, topological approaches to attack tolerance are needed to address the current vulnerability of communications infrastructure to censorship and surveillance. We present a novel concurrent multipath routing (CMR) algorithm for the wraparound butterfly network topology, as well as a highly attack-tolerant Structured Multipath Fault Tolerance (SMFT) architecture which incorporates the butterfly CMR algorithm. We also identify a previously unexplored relationship between network topology, trust transitivity, and attack-tolerance, and provide a framework for further exploration of this relationship. Our work is the first theoretical demonstration of a point-to-point communication network architecture that can resist coercion and other non-technical attacks, without requiring infinitely transitive trust. To address cases where the network structure cannot be fully controlled, we demonstrate how a snapshot of the internet’s router network can be partially rewired for greater attack-tolerance. More broadly, we hope that this work will serve as a starting point for the evelopment of additional topology-based attack-tolerant communication architectures to guard against the dangers of censorship and surveillance.

## Introduction

Is it possible for any large-scale communication network to resist targeted attacks? The internet was originally designed to withstand targeted (nuclear) attacks [1], and the resilience of the internet has long been part of common wisdom [2]. But 18 years after Albert et. al [3] showed that the topology of the internet’s router network makes it vulnerable to targeted attacks (vs. random faults), the fundamental problem of attack-tolerant network topology remains unsolved. Attack-tolerant topologies are desirable not only for the router network, but for any physical or virtual network where a compromised node puts communication at risk. For example, the network of verified keys in the public key infrastructure underlying secure http [4], or the network of DNS nameservers. The ongoing vulnerability of the internet is evidenced by a long history of censorship and surveillance incidents achieved by means of targeted attacks [5]. In this paper, we present the first theoretical network topology supporting attack-toloerant, point-to-point networked communication, without relying on infinitely transitive trust [6].

Methods for tolerating various kind of faults within networks are an important and ongoing area of research [3, 7, 8]. *Adversarial faults*, those in which an adversary can target attacks strategically, deserve special attention. Such attacks are both extremely difficult to guard against and often have important social implications. In particular, censorship and surveillance are often achieved by targeting central network locations and either blocking or capturing the information flowing through them. While cryptograpy can provide some protection against surveillance, it is vulnerable to *man-in-the-middle* attacks [9], and cannot overcome censorship when communication is blocked. In this paper, we instead consider a topological approach. The Internet’s decentralized design was motivated by the need to withstand targeted attacks, such as nuclear strikes [1]. But despite longstanding common wisdom [2], both theoretical results and recent events have demonstrated that the internet is surprisingly vulnerable to attack.

Analysis of the internet’s router network has shown that while it is remarkably resilient against random faults, it is highly susceptible to adversarial faults [3]. These results have been attributed to the heavy-tailed degree distribution of the Internet’s router network [10, 11]. Random failures are highly likely to affect only low-degree nodes, thus having little effect. However, adversarial faults target the few high-degree nodes, and therefore remove a large number of edges with each fault. So while the *protocols* of the Internet are decentralized, the *network structure* is somewhat centralized. In other words, the protocols of the Internet do not *require* centralization, but centralization may still emerge from the sociotechnical processes that create its network structure.

The internet’s vulnerability to censorship and other targeted attacks has been demonstrated by several recent events. In 2008, YouTube suffered a worldwide outage for several hours when a service provider in Pakistan advertised false routing information [12]. The action (known as a *black hole attack*) was intended to censor YouTube within Pakistan only, but resulted in a worldwide cascading failure when a router misconfiguration allowed the false routing information to propagate outside of Pakistan. This incident exemplifies the type of attack requiring a topological approach. First, the attack was *non-technological* (a government order), allowing the attacker to bypass any cryptographic or technology-based defenses. Second, the attack originated at a *single point of failure* (a misconfigured router). Third, the behavior of the compromised component (the router) cascaded through a *network* (the network of internet routers) because the correct behavior of other components depended on the correct behavior of the single point of failure. And while the action was not an intentional attack against the global internet, the ability of an attacker to succeed without even trying only highlights the internet’s vulnerability to adversarial faults.

As another example, in 2013, the Texas-based email provider Lavabit was ordered to disclose their private SSL keys to the FBI [13]. Rather than complying, Lavabit ceased operations in order to protect their users from surveillance. Once again, the attack was non-technical. And again, the attack was on a single point of failure: Lavabit’s web server and that server’s TLS/SSL keys. In this case, the affected network was the internet’s public key infrastructure. With the private keys, an attacker would be able to intercept and surveil traffic because the issuing certificate authority (and any users trusting that authority) would incorrectly trust that they were communicating with Lavabit. While originally intended as surveillance, this action effectively became an act of censorship. So we see that such vulnerabilities are not limited to any one system or protocol, but result from centralized structure itself.

This paper addresses the need for a theoretial understanding of network and redundancy-based approaches to attack-tolerance. For the purpose of this paper, *attack-tolerance* refers to the ability of a pair of communicating nodes to detect, with high probability, when a message has been blocked or altered in the presence of adversarial faults. Our primary result is theoretical: an algorithm for constructing highly redundant paths in a particular network topology. While we motivate the need for such network topologies using examples such as the internet router network and webs of trust, we do not propose that our algorithm as a pracitcal solution for any of these examples. Rather, our result is a demonstration that such topological approaches to attack-tolerance are theoretically possible and suggest the importance of further theoretical and applied work.

We consider a setting in which a source node attempts to route a message to a target node, while an adversary attempts to block or intercept the message by compromising a number of intermediate nodes. We also assume that edges represent *direct trust*, i.e., the belief that an adversary is unlikely to compromise a neighbor, as in the web of trust approach [14, 15]. The web of trust approach typically assumes infinite trust transitivity in that it is possible for one node to have some level of trust for another as long as they are connected by a path of directly trusted edges, regardless of the length of that path. The assumption of infinite transitivity is unrealistic [6], so we instead make a stricter assumption: *bounded trust transitivity*, that trust transitivity can only be extended over a finite number of edges. Even with this more restrictive assumption, we show how trust can be established between two nodes even when no path of directly trusted edges exists between them.

Under the above assumptions, we show how to evaluate the influence of network structure on attack-tolerance. We next present a structured multipath fault tolerance (SMFT) scheme to extend standard fault tolerance techniques to the case of adversarial faults in networks [16, 17]. The SMFT scheme requires the existence of a concurrent multipath routing (CMR) algorithm [7, 18, 19], to takes advantage of the independence of faults along *independent paths*. We also present a novel CMR algorithm for the butterfly network topology. The butterfly topology is popular in parallel processing [20] and peer-to-peer [21, 22] applications, due to its regular structure, low degree, and high connectivity.

It is important to note that the butterfly is a highly structured and constrained network topology, very different form those found in social networks and other self-organized networks. The reader may wonder whether it is realistic or useful to assume such control over the network structure. Regardless of implementation difficulty, we argue that attack-tolerance cannot be achieved without the ability to influence network structure. Targeted attacks, by definition, target single points of failure. Attack tolerance can thus be achieved in two ways: 1. preventing failure at those points, or 2. preventing the existence of central points. Because individual points are always vulnerable to coercive, non-technological attacks, the former method is insufficient. We must instead rely on some control over topology to prevent the existence of single points of failure. The difficulty of achieving control over network topology can be mitigated by a number of approaches. Attack-tolerant networks might be sub-components of larger, less-constrained systems. For example, a single centralized server might be replaced by a distributed network of servers, each with different ownership, physical location, and legal jurisdiction, without placing any unrealistic constraints on the clients connecting to those servers. When attack tolerant topologies are nested (as in the case of the butterfly topology), multiple independent sub-components could be merged into larger ones over time. Real-world examples of structured neworks include: overlay networks [21, 22], formal organizations [23], government-regulated cellular networks [24], and call tree notification systems [25]. In general, when the need for attack-tolerance is high enough to warrant investment in infrastructure, topology can be engineered and maintained as infrastructure.

Our main contributions are:

- We propose a novel structured multipath fault tolerance (SMFT) scheme for extending standard fault tolerance techniques to *adversarial* faults in *complex networks*. Assuming *h*-degree *bounded trust transitivity*, We show that the probability of detecting adversarial faults *h*-internally vertex disjoint paths.
- We prove that the number of *h*-internally vertex disjoint paths between two nodes in a directed wrap-around butterfly network is exactly 2^*h*^, and present a scalable and efficient concurrent multipath routing (CMR) algorithm to find these paths, which can be combined with SMFT to achieve a high level of attack-tolerance.
- We show that rewiring a the edges of the internet’s router network to resemble a butterfly network allows it to tolerate a higher number of failures without fragmenting, and increases the effective redundancy in the presence of a large number of adversarial faults.

This paper is organized as follows. Section reviews background and related work. Section describes adversarial fault tolerance on structured networks. Section gives background on the butterfly network topology. Section presents our concurrent multipath routing algorithm for the butterfly network. Section discusses the results. And Section concludes.

## Background and related work

There has been considerable work on trust-based attack-tolerance techniques in network security, both centralized and decentralized. Centralized approaches such as *public key infrastructure* (PKI) suffer from a number of vulnerabilities [4], including vulnerability to coercion, which stems largely from the single points of failure inherent to centralization. The well-known and widely-used *web of trust* approach [14, 15] is a decentralized alternative. In a web of trust, individuals can have *direct trust*, as well as *indirect trust* for those trusted by someone directly trusted. Typically, this transitivity is extended to any number of hops, sometimes reducing trust by a multiplicative factor at each hop. Infinite trust transitivity is helpful for establishing a large group of trusted nodes, but unfortunately unrealistic [6]. Our work addresses this limitation by assuming only bounded trust transitivity.

Previous work applying network topology to attack tolerance has focused on authentication, showing that independent paths can reduce an adversary’s ability to impersonate a target [26]. Other work has shown that identifying independent paths in arbitrary networks is NP-hard and provided approximation algorithms [27]. Our work complements these results by extending our focus beyond authentication, to communication. When network topology can be controlled, we sidestep the NP-hard problem of finding independent paths on arbitrary networks by using the mathematical structure of the butterfly topology to construct provably independent paths.

Many distributed consensus protocols (such as those used by cryptocurrencies) are designed to tolerate arbitrary or adversarial faults. Byzantine agreement protocols [28, 29] provide tolerance against arbitrary faults (including attacks) under some circumstances, but are limited to small networks due to poor scalability. Proof-of-work [30, 31] (blockchain) systems provide better scalability, but are wasteful of computational and energy resources, and do not take advantage of trusted relationships. Federated Byzantine Agreement (FBA) [32] is scalable, allows for flexible trust, and is highly fault-tolerant on networks meeting specific requirements. However, FBA does not provide a method for constructing networks to meet those requirements, or for calculating the failure probabilities within a particular network.

All existing attack-tolerant networks we are aware of are content-addressable networks (CANs) in which data is stored and retrieved based on key values, rather than point-to-point networks, in which data is communicated between two parties. Fiat and Saia described a scheme that combines the butterfly topology with expander graphs to create a highly censorship-resistant, content-addressable network [33], although this scheme requires high levels of data replication and indefinite storage. Perhaps the most mature structural solution is the Freenet collaboration [34]. Freenet uses secret sharing [35, 36] and small-world routing [37, 38] to create a content-addressable network with a high level of both confidentiality and censorship resistance. Freenet guarantees that data is stored redundantly, but still allows for centralized network structure, and thus single points of failure, as data travels from its origin to the redundant storage locations. Unlike the above content-addressable networks, our architecture is purely network based and does not require nodes to store data indefinitely. Our architecture also improves on the scalablity of the Fiat-Saia network, and makes requirements about network topology explicit.

*Multipath routing* protocols identify multiple paths between source and destination in contrast to traditional *unipath* routing, which uses a single path. The special case of *concurrent* multipath routing uses multiple paths simultaneously. Multipath routing has many applications, including reduced congestion, increased throughput, and more reliability [18]. Many of these routing protocols offer increased confidentiality [7]. Some approaches utilize redundant paths as backups for increased fault tolerance [39], and some specifically protect against adversarial faults [40–42]. Most work on multipath routing has been motivated by applications related to wireless sensor networks (WSNs), and have thus focused on ad-hoc, unstructured networks, often having a central base station. The method of Liu et al. [43] routes multiple messages first to random peers and then to a central base station, with the network edges constrained by sensors’ physical location. We have found very few examples of CMR applied to *adversarial* fault tolerance in the existing literature, and all have focused on ad-hoc wireless sensor networks, without attention to the role of network structure.

Our proposed routing algorithm makes use of a *structured network*, in which link structure is predetermined. Structured networks have been a popular tool in parallel processing architectures [20]. More recently, peer-to-peer systems based on distributed hash tables have used structured *overlay networks* to map table keys to local TCP/IP routes [21, 22]. Such networks can be designed to have favorable structural and routing properties, which can be used to to improve attack-tolerance.

Our proposed architecture is differentiated from existing systems by several properties (Table 1). Decentralized architectures are more resistant to coercion [44] and man-in-the-middle attacks [9]. Trust-based systems are more sustainable than proof-of-work. Bounded-trust systems do not require the unrealistic assumption of infinite trust transitivity. Topological approaches address the root cause of vulnerability in heavy-tail networks, rather than relying on technology that can be side-stepped through coercion. Point-to-point communication allows two individuals to exchange messages without requiring large amounts of indefinite data storage on intermediate nodes.

**Table 1:** Comparison of attack-tolerant network communication architectures.

	Decentra-lized	Trust-based	Bounded-trust	Topo-logical	Point-to-point
PKI		✔	✔		✔
Web of Trust	✔	✔			✔
Freenet	✔	✔	✔		
FBA	✔	✔	✔		✔
Proof of Work	✔		✔		
Fiat-Saia	✔	✔	✔	✔	
SMFT	✔	✔	✔	✔	✔

## Trust networks and fault tolerance

Within the field of *fault tolerance*, many techniques have been developed for building reliable systems out of unreliable components [16, 17]. We will make use of standard fault tolerance terminology, summarized here. A *fault* is occurs when one component of a system behaves incorrectly (e.g., a routing node blocks or alters a message). The result of that fault (e.g., a recipient receiving conflicting messages) is an *error* state. If the error is undetected or corrected to the wrong value, the system has experienced a *failure* (e.g., an altered message is accepted as authentic). Note that when an error is detected but cannot be corrected, the system has still tolerated the fault because it has not accepted an error state. We are concerned in particular with *adversarial faults*, which are chosen strategically to maximize the likelihood of a failure.

### Multipath fault tolerance

Standard fault tolerance methods use redundancy to detect and correct statistically independent faults. In complex networks however, faults can be correlated when, for example, two messages pass through the same faulty node. For now, let us assume our sender (Alice) and receiver (Bob) are connected by *δ* direct channels, with independent errors. We will return to the question of constructing these channels in subsequent sections. For now, we concern ourselves with the question: given that the network provides *δ* redundant channels between Alice and Bob, what is the probability that an adversary (Mal) causes an undetectable error after causing faults in a fixed number of channels?

Let us first consider the scenario in which Alice sends a message copy over each available channel. We can also assume that each message includes the number of messages sent, the full list of channels used, etc., making that information available to Bob. When Bob receives the messages, there are several possibilities. If some of the messages are missing or if some of the messages disagree, Bob knows that some of the messages were either blocked or altered, and he has successfully tolerated the fault(s). Bob can then take any of several actions: 1. request re-transmission; 2. send receipts so Alice knows which paths have been compromised; or 3. attempt error correction using majority voting. If instead, Bob finds that all the messages are present and agree, there are two possible cases. The first case is that Mal has not compromised any of the messages, and Bob has correctly accepted them, so no failure has occurred. The second case is that Mal has compromised *all* of the messages, so Bob has accepted an erroneous message and a failure has occurred. In the present scenario, whether a failure occurs depends only on whether Mal has the resources to compromise all of the channels. In a more realistic scenario, both Alice and Mal have limited resources and are not able to use or compromise all available channels.

In a more sophisticated multipath fault tolerance scheme, Alice randomly chooses *k* ≤ *δ* channels and sends a copy of her message on each. We assume that Mal is capable of compromising *l* ≤ *δ* channels. Since Alice chooses channels randomly, all channels are equally likely to contain a message, so Mal can do no better than also choosing randomly. If *k* > *l*, at least one message will get through uncompromised and all errors are detectable. Otherwise, the probability of Mal producing an undetectable error is the probability that all of Alice’s chosen channels are compromised:

$$p_{f}=\frac{l!(\delta -k)!}{\delta !(l-k)!}.$$ (1)

Letting *k* = *αδ* and *l* = *βδ*, then applying Stirling’s approximation gives:

$$\begin{array}{ccc} p_{f} & \approx & \frac{\sqrt{\beta (1-\alpha )}}{\sqrt{\beta -\alpha }}[(\frac{\beta -\alpha }{1-\alpha }{)}^{\alpha }(\frac{\beta }{\beta -\alpha }{)}^{\beta }\left(1-\alpha \right){]}^{\delta }. \end{array}$$ (2)

**Fig 1:**
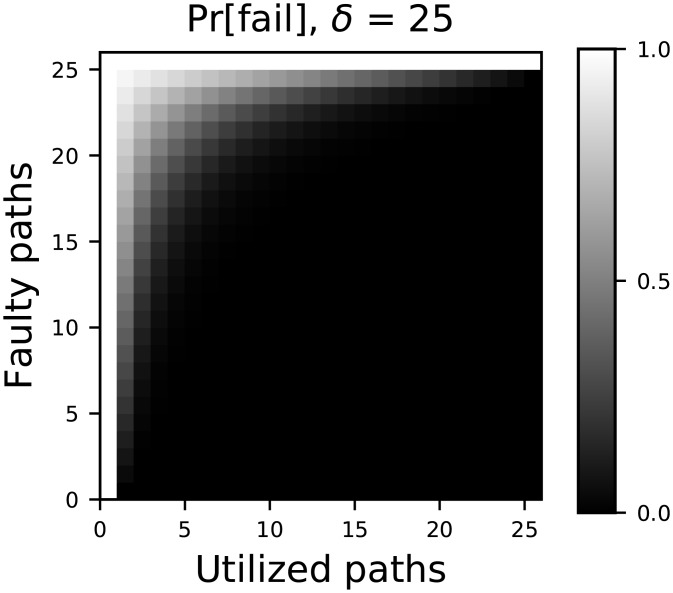
The probability of an undetectable error as a function of the number of redundant channels and the number of adversarial faults. A small increase in the number of utilized paths (network traffic) can compensate for a large increase in attacker power.

Fig 1 shows the value of *p*_*f*_ as a function of *k* and *l*. Eq (2) shows that while *p*_*f*_ depends on the fractions of channels actually utilized *α* and compromised *β*, it decreases exponentially with *δ*. This result is significant because, as we will soon show, *δ* depends only on the network structure and the strength of trust transitivity. *Thus, the scheme can be effective, even when the number of channels used k is a small fraction of the channels available*. In other words, this scheme exhibits a *stabilizing asymmetry*: senders can tolerate attacks from significantly more powerful adversaries. Furthermore, this scheme requires only a small increase in network traffic as long as the network provides a large number of independent paths *δ*.

### Bounded trust model

So far, we have assumed that Alice and Bob have access to some number *δ* of channels with statistically independent faults. However, in real communication architectures, direct links between all pairs of individuals are not possible and messages must be routed through a number of intermediate nodes. In an adversarial setting, the existence of intermediate nodes introduces two problems: 1. intermediate nodes may be compromised by the adversary and 2. faults on paths are no longer statistically independent: two paths may pass through the same compromised node. We show how to how to resolve these problems using a combination of network structure and bounded trust transitivity.

Trust-based approaches to secure communication assume that some parties cannot be compromised. One common approach, the web of trust [14, 15]. Alice has *direct trust* for some number of nodes. Alice has *indirect trust* for nodes separated by two or more hops of direct trust. Typically, this transitive trust is extended indefinitely, sometimes reduced by a multiplicative factor at each step. However, the assumption of infinitely transitive trust is unrealistic [6]. Furthermore, infinite trust transitivity obscures the importance of network structure, as it depends only on whether some path exists, not the number or quality of paths. We adopt a simpler, yet more realistic *bounded trust* assumption: that nodes up to some fixed number of hops cannot be compromised, and that those beyond can. This assumption will be convenient for proving our results, which we now proceed to define formally.

**Fig 2:**
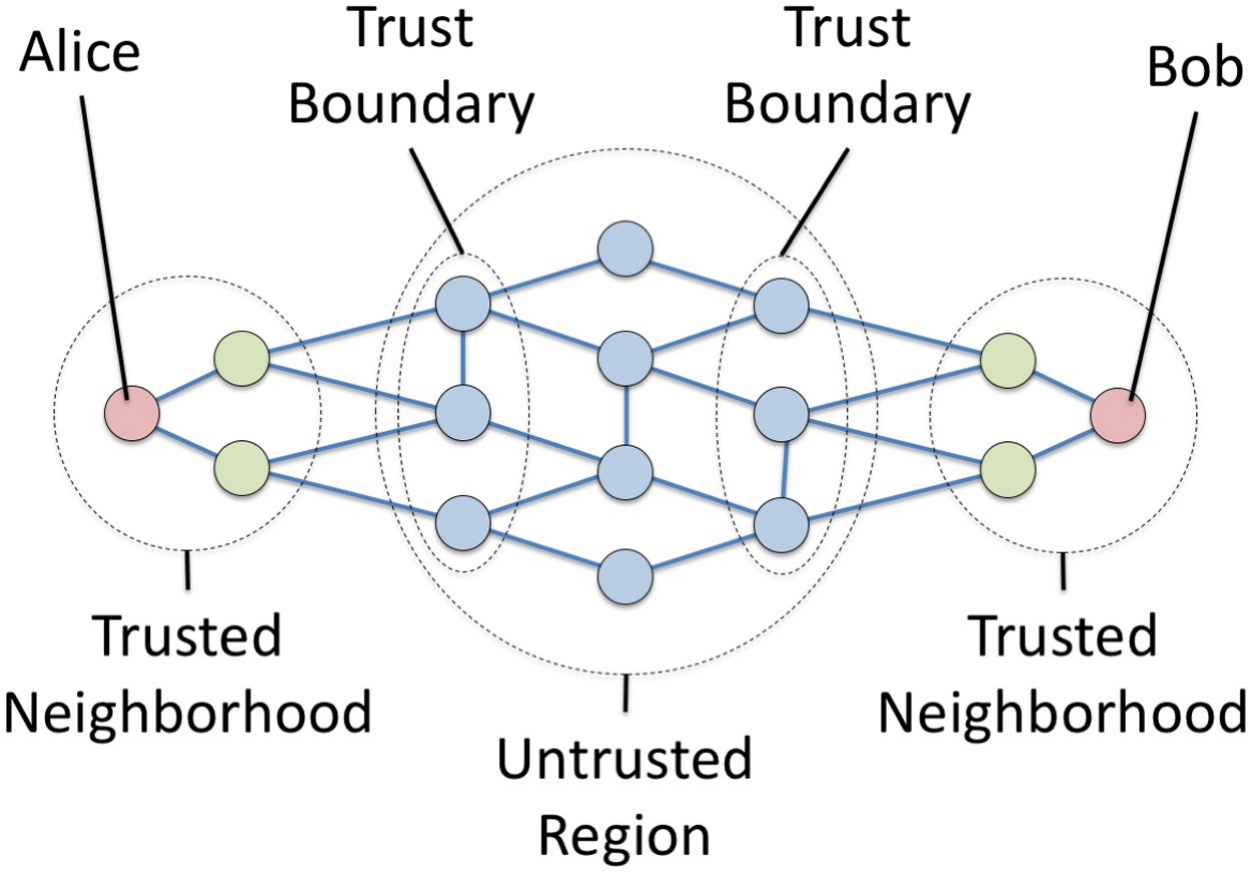
Illustration of a trusted communication network and the network properties used by the *bounded trust model*. Edges represent mutually trusted communication links. The sender (Alice, *s*) and receiver (Bob, *t*) trust all nodes less than the *trust radius* *h* hops away. These nodes form their *trusted neighborhoods T*_*h*_(*v*) and *T*_*h*_(*w*). We assume that all faults occur in the remaining nodes: the *untrusted region*. The untrusted nodes in contact with the trusted neighborhoods for the *trust boundaries B*_*h*_(*v*) and *B*_*h*_(*w*), which (in the absence of central bottlenecks) determine the *effective redundancy δ*_*h*_ provided by the network. Alice and Bob can achieve the same level of attack-tolerance as if they were directly connected by *δ*_*h*_ redundant channels.

We define the *bounded trust model* (Fig 2) on an undirected graph *G* = (*V*, *E*), although the model can easily be extended to directed multigraphs. Vertices representing communicating parties, with edges representing mutually trusted communication links. We define a *trust radius* *h* such that nodes *v* and *w* trust each other if their distance is less than *h*. For a given node *v*, we call the set of trusted nodes its *trusted neighborhood T*_*h*_(*v*), and all nodes at exactly distance *h* the *trust boundary B*_*h*_(*v*):

$$T_{h}\left(v\right)=\{w|d(v,w)<h\}$$ (3)

$$B_{h}\left(v\right)=\{w|d(v,w)=h\}.$$ (4)

The trust boundary *B*_*h*_ plays an important role because these nodes are not trusted by *u*, and if compromised can entirely isolate *v* from the rest of the network.

Now let *s* ∈ *V* be an arbitrary sender and *t* ∈ *V* be an arbitrary receiver. We assume the presence of an adversary who knows the full structure of the network, and who can compromise a fixed number of nodes, gaining complete control of their behavior. We also assume that the adversary is specifically targeting communication between *s* and *t* and can compromise any node except for those trusted by *s* or *t*. Under these trust assumptions, adversarial faults can only occur outside the trusted neighborhoods of *s* and *t*: *V*\(*T*_*h*_(*v*) ∪ *T*_*h*_(*w*)). We refer to this set of nodes as the *untrusted region*. We now show how it is possible to communicate reliably, even when all available paths go through the untrusted region.

### Effective redundancy

Our approach is to achieve fault tolerance through redundancy. To do so, we must use only *independent paths* [27], which have no common points of failure. Typically, it is assumed that in order to be independent, paths must be internally vertex disjoint, i.e., have no nodes in common except the endpoints. However, under the bounded trust model, intersecting paths can still be independent if their intersection contains only trusted nodes. We define two paths with common endpoints to be *h*-*internally vertex disjoint* if all common vertices are less than distance *h* from one of the endpoints. This condition holds if and only if two paths are independent under the bounded trust model with radius *h*.

When trust radius *h* is assumed, the number of *h*-internally vertex disjoint paths between two nodes *s* and *t* represents the number of channels that can be constructed between them having statistically independent faults. We thus refer to this quantity as the *effective redundancy δ*_*s*,*t*,*h*_. The effective redundancy can also be interpreted as the max-flow/min-cut of a graph after each trusted neighborhood has been collapsed into a single vertex. The trust boundaries form a cut of the network and place an upper bound on the min-cut:

$$\begin{array}{c} \delta_{v,w,h}\leq \min(|B_{h}\left(s\right)|,|B_{h}\left(t\right)|). \end{array}$$ (5)

Equality holds when there are no bottlenecks within the untrusted region, an indication that the network is decentralized. The effective redundancy of the entire graph can be characterized by the minimum over all vertex pairs:

$$\begin{array}{c} \delta_{h}\left(G\right)\equiv \min_{s,t\in V}\delta_{s,t,h}. \end{array}$$ (6)

Thus, for any pair of nodes in the network, at least *δ*_*h*_ independent, redundant paths can be constructed between them. The more quickly *δ*_*h*_ grows with *h*, the better a network is at leveraging trust transitivity to create redundancy. Thus, the scaling of *δ*_*h*_ can be used to quantify a network’s ability to withstand targeted attacks, even when the exact trust radius *h* is unknown.

### Structured multipath fault tolerance

Finding a maximal set of independent paths for an arbitrary network is NP hard [27], posing a challenge for multipath fault tolerance. We propose side-stepping this problem by using structured networks, for which independent paths can be generated efficiently. We call this approach *structured multipath fault tolerance* (SMFT), and now proceed to show how it is implemented on the butterfly network topology.

## The butterfly network topology

In order to implement structured multipath fault tolerance, we need a structured network topology with high effective redundancy. In this paper, we apply SMFT to the butterfly network topology [20]. The butterfly network is highly structured, making it most suitable for applications where portions of the network structure can be controlled or influenced. The butterfly network is also recursive, with larger versions composed out of multiple smaller versions, making it possible for independently organized attack-tolerant networks to merge into larger ones over time. More flexible architectures may be possible, but attack-tolerance will always require some level of influence over network structure in order to limit single points of failure. To address the case when the network cannot be fully controlled, we show how partially rewiring a snapshot of the internet’s router network can greatly increase it’s effective redundancy and attack-tolerance properties, without requiring additional edges.

### Butterfly network topology

**Fig 3:**
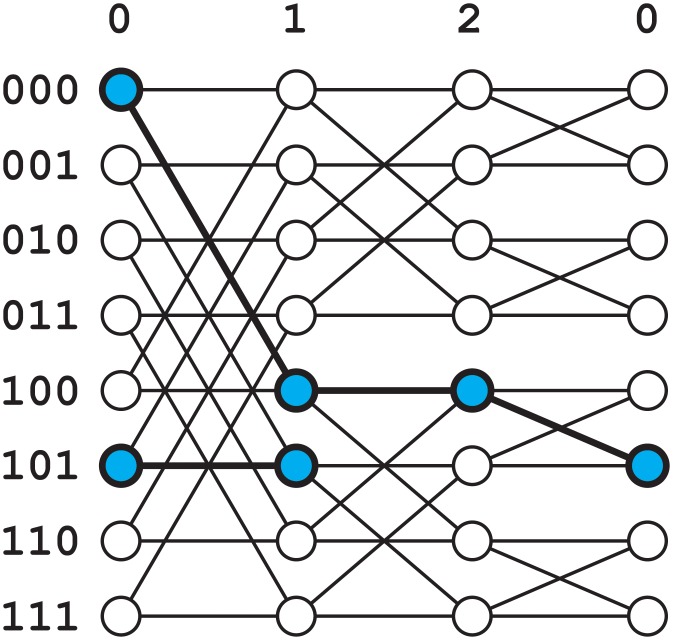
A 3-dimensional wrap-around butterfly network. Note that the rightmost nodes are the same nodes as the leftmost, drawn twice for visual clarity. The highlighted nodes and edges show the path from node (0,000) to node (1,101).

**Fig 4:**
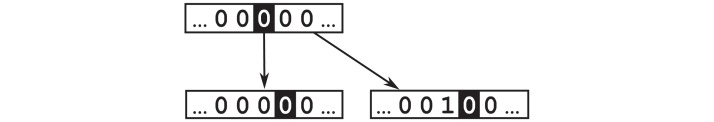
Schematic illustration of the two types of edges in a directed butterfly network. The node (*l*, *z*) is shown as the bit string *z* with a square around the *l*th bit. “Down” edges increment *l*, leaving *z* unchanged, while “down-right” edges increment *l* and invert the *l*th bit of *z*. In the wrap-around variant, the nodes with maximum *l* have down and down-right edges to the nodes with *l* = 0.

We choose the butterfly topology [20] because of several desirable properties (described below) and because its structure allows for relatively straightforward design and analysis of routing algorithms. While several variations on the butterfly network exist, we utilize the *m*-dimensional, directed wrap-around butterfly (Fig 3), denoted wBF(*m*):

$$\mathrm{wBF}(m)=\left(V,E_{↓}\cup E_{\to }\right)$$ (7)

$$V=Z_{m}\times Z_{2}^{m}$$ (8)

$$E_{↓}=\left\{((l,z),(l+1\;(\text{mod}\;m),z)\right\}$$ (9)

$$E_{\to }=\{\left(l,z\right),\left(l+1\;\left(\text{mod}\;m\right),z⊕1_{l}\right\},$$ (10)

where $Z_{m}$ is the set of integers modulo *m*, ⊕ represents component-wise addition modulo 2, and 1_*l*_ is a vector with a 1 in index *l* and 0 elsewhere. Each node is associated with a level *l* and an *m*-bit string *z* known as *the place-within-level*. There are two types of edges: down, and down-right (shown in Fig 4). Down edges (*E*_↓_) connect nodes sharing the same *z* value in a cycle of increasing level *l*. Down-right edges (*E*_→_) also link to a node of level *l* + 1, but one having the place-within-level equal to *z* with the *l*th bit inverted.

The wrap-around butterfly network is known to have several of the properties we desire for scalable, decentralized communication networks:

**Vertex-transitivity**: Because the wrap-around butterfly is vertex transitive, it is maximally decentralized;
**Small-diameter**: For any two nodes, the length of the shortest path between them is *O*(log *N*), where N is the number of nodes in the network (corresponding to low-latency in real-world terms);
**Sparsity**: With a constant degree of 4, the wrap-around butterfly is extremely sparse, and can scale indefinitely without node degree becoming a limitation;
**Redundancy**: Multiple paths exist between any two nodes. Specifically, we will prove below that the number of *h*-internally vertex disjoint paths between two nodes increases exponentially with *h*.

The structure of the butterfly network lends itself to a well-known (unipath) routing algorithm (Fig 3), which we later extend to the multipath case. The unipath algorithm first follows a down or down-right edge at every step, increasing the level *l* by 1 and cycling through the indices of the place-within-level. If the current node’s place-within-level matches the destination node’s at index *l*, a down edge is chosen and the place-within-level does not change. Otherwise, a down-right edge is chosen and the *l*th component of the place-within-level is flipped, after which it matches the destination. After *m* iterations of this, all levels have been visited and the place-within-level matches that of the destination. Simply following down (or up) edges will then increment (decrement) the level until the destination node is reached.

### Butterfly rewiring

Even when a butterfly topology cannot be implemented perfectly, it can still increase the attack tolerance properties of a network. Here, we simulate targeted attacks against a snapshot of the internet’s router network on January 2, 2000 [45], having 6493 nodes and 13914 edges. At each step of the simulation, betweenness centrality is recalculated and the most central node is removed. We also simulate attack on several rewired networks. The rewiring process alters the network structure to resemble a butterfly topology, without adding any additional edges. We 1. generate edges corresponding to a 9-dimensional butterfly network between the 4608 highest-degree router nodes, 2. choose a fraction *f* of those edges at random, 3. add those edges to the router network, and 4. remove an equal number of the original edges at random.

**Fig 5:**
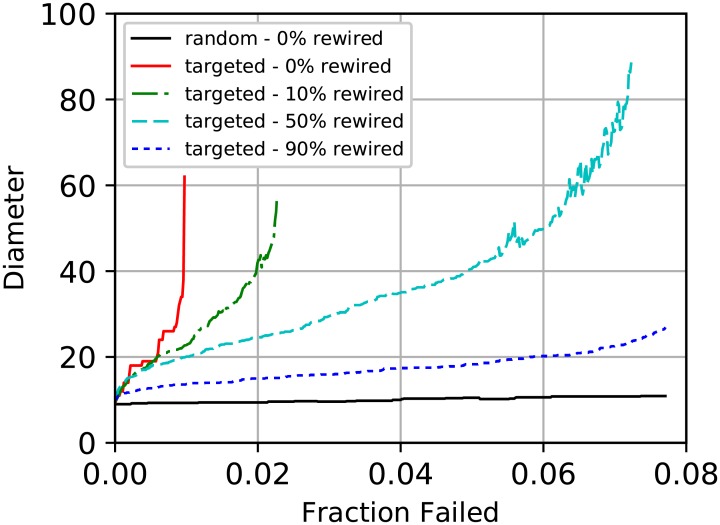
Simulation of targeted attacks against a snapshot of the internet’s router network with a fraction of the edges rewired into a partial butterfly configuration. The original network fragments when the top 1% of nodes are removed. With only 10% of the butterfly edges present, this value doubles to 2%. With 90% of the edges rewired, the network remains unfragmented beyond the failure of the 8% most central nodes. The rewiring scheme does not require adding any additional edges.

**Fig 6:**
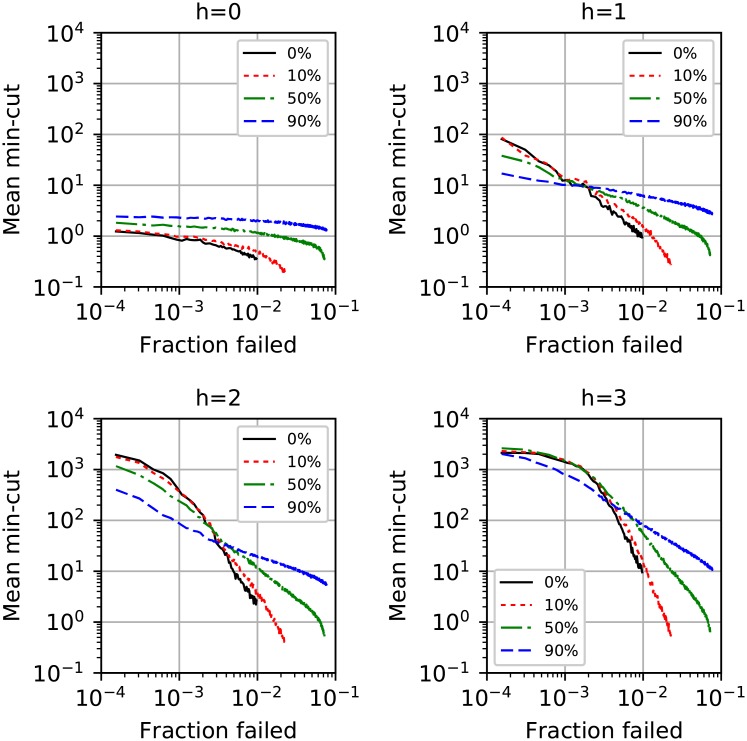
Simulation of targeted attacks against a snapshot of the internet’s router network with a fraction of the edges rewired into a partial butterfly configuration. The effective redundancy is shown for several values of trust transitivity *h*. For *h* > 0, the original network has higher effective redundancy up to a crossover point, after which the rewired network performs better.

Our simulations show improved resistance to fragmentation and higher effective redundancy when even a fraction of edges have been rewired to match the butterfly topology. While the original router network fragments when about 1% of the nodes have been removed (Fig 5), this number increases to 2% with only 10% of the butterfly edges present. With 90% of the butterfly edges present the network remains unfragmented beyond the failure of the 8% most central nodes. The effective redundancy for various values of trust transitivity *h* are shown in Fig 6. The effective redundancy is calculated by collapsing nodes and edges within *h* hops of source and destination into single nodes and finding the min-cut between them, averaging over 150 source-destination pairs. For *h* = 0, the rewired version has strictly higher redundancy. For *h* > 0, the original network has higher redundancy when the number of failures is small, while the rewired network has higher redundancy beyond a crossover point. We interpret these results to suggest that even when a small number of highly central nodes have been removed from a heavy-tailed network, most nodes are still able to take advantage of the remaining hubs. As larger hubs continue to be removed, the connectivity of the network decreases until the crossover point, at which point the rewired network offers higher effective redundancy.

## Multipath butterfly routing

We now present a routing algorithm to construct 2^*h*^ independent paths between two nodes in a butterfly network, where *h* is the trust radius under the partial trust model. Informally, Alice sends each message to a distinct node on her trust boundary, then to a distinct intermediate node in the untrusted region, then to a distinct node on Bob’s trust boundary, and finally to Bob. The intermediate nodes are in a sense “far” from each other and ensure that no two paths overlap in the untrusted region. Each path can be parameterized by a single integer *s*, which identifies the specific node on Alice’s trust boundary (or equivalently the node on Bob’s trust boundary, or the untrusted intermediate).

**Fig 7:**
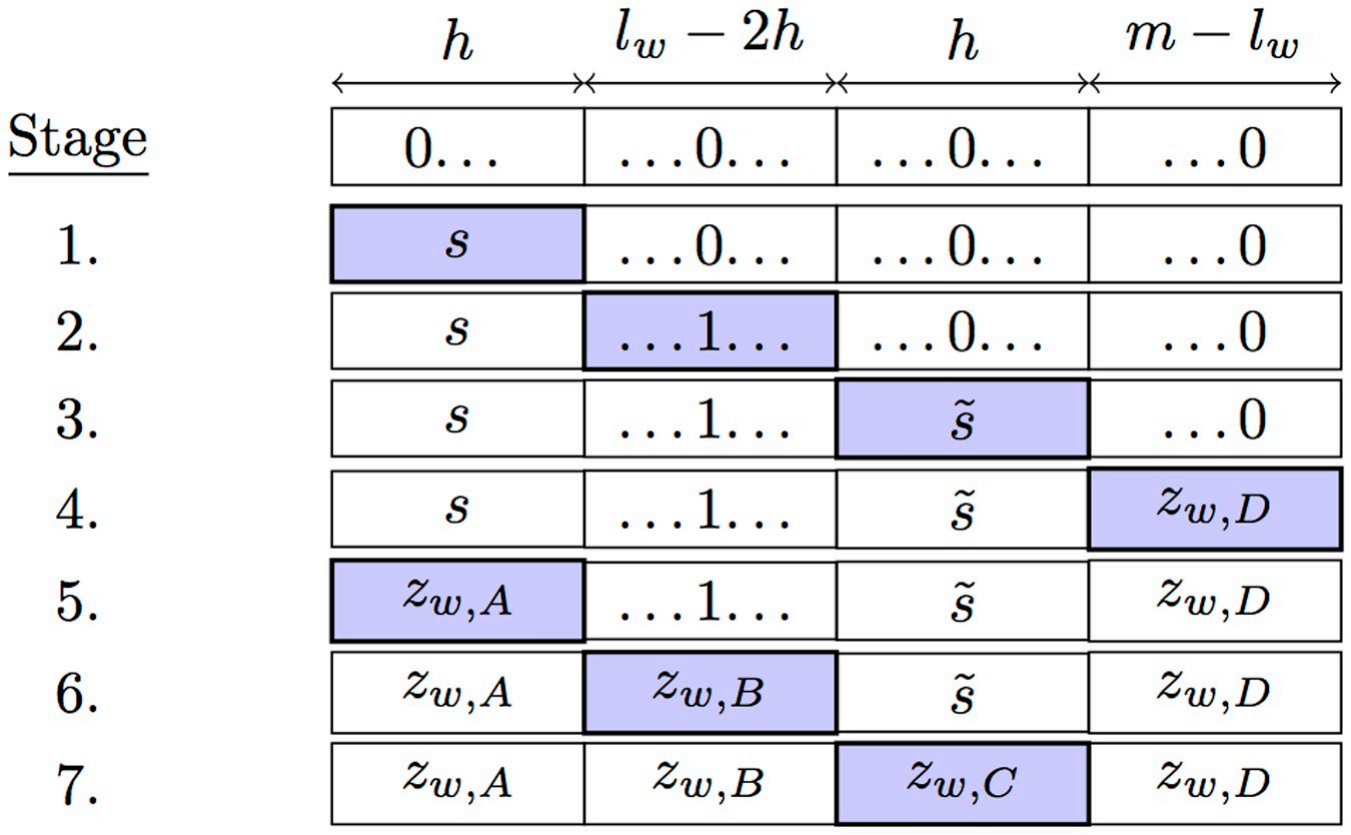
Progression of place-within-level *z* as the multipath routing algorithm cycles through the levels of the butterfly network.

**Fig 8:**
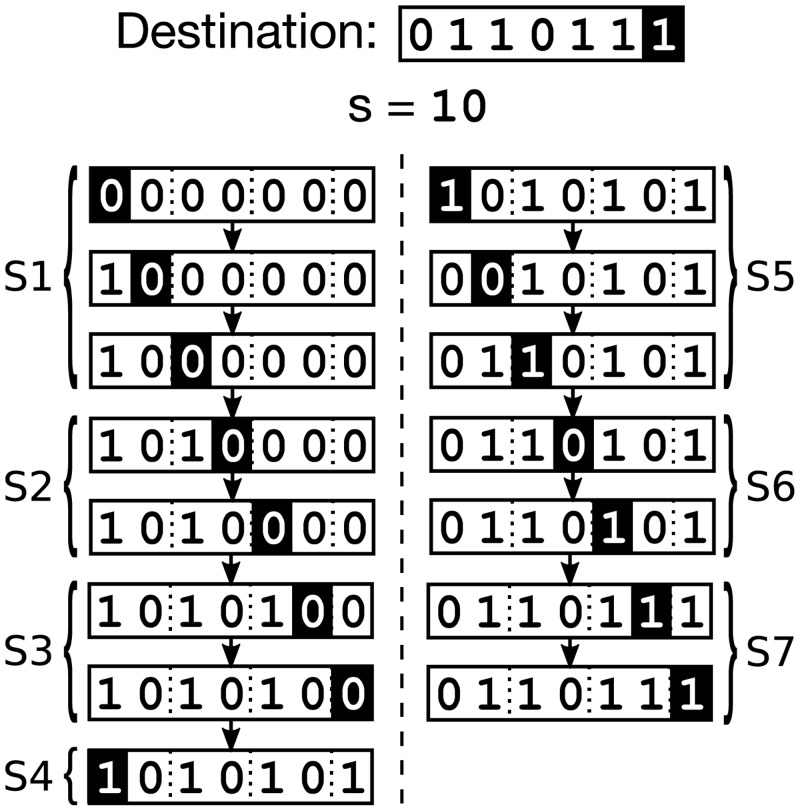
An example of one path as constructed by the proposed multipath routing algorithm. The path is shown for *s* = 10_2_ and *w* = (6, 0110111_2_).

The algorithm guarantees paths are independent by ensuring that (outside the trusted neighborhoods) they only include nodes that match the path parameter *s* at certain indexes in their place-within-level. Since each path has a unique parameter *s*, its set of untrusted nodes is disjoint from all other paths. As with the unipath routing algorithm, each of the multiple paths proceed from a source *v* to a destination *u* using down and down-right edges, cycling through levels one at a time. However, we cycle through the levels twice, once to route from *v* to a particular path’s intermediary node, and again to route from the intermediary to *w*. Each cycle is divided into stages, with different properties used to prove independence at each stage (see Fig 7). In the first cycle (stages 1–4), path independence is guaranteed by ensuring that all nodes match the path parameter *s* in the first *h* bits of the place-within-level. Similarly, in the second cycle (stages 5–7), independence is guaranteed by ensuring that all paths match *s* in the *h* bits of the place-within-level preceding the destination index. A full example is illustrated in Fig 8.

### Algorithm specification

We now begin the formal specification of our multipath routing scheme for the wrap-around butterfly network. For convenience, the relevant variables are summarized in Table 2. Utilizing vertex transitivity, we label the source node as (*l*^(0)^, *z*^(0)^) = (0, 0) and denote the destination node as *w* = (*l*_*w*_, *z*_*w*_), without loss of generality.

**Table 2:** Butterfly multipath routing variables.

Name	Variable
butterfly dimension	$m\in Z_{+}$
node level	l∈Z:0≤l<m
node place within level	$z\in Z_{2}^{m}$
trust radius	h∈Z:1≤h≤⌊m/2⌋
path index	$s\in Z_{2}^{h}$

Let *s* be an *h*-bit binary string with *s*_*i*_ denoting the bit at index *i*. There are 2^*h*^ such strings. Let $v_{s}^{\left(t\right)}=\left(l^{\left(t\right)},z^{\left(t\right)}\right)$ be the node at position *t* in the path parameterized by *s*. For convenience, we will omit the subscript *s* when it is obvious from context. We define three distinct partitions of *m*-bit binary strings. Let $Q_{v^{\left(0\right)}}$ be the set of *m*-bit strings in which the bits at all indices *h* ≤ *i* < *l*_*w*_ − *h* match those of *z*^(0)^, and let $\bar{Q_{v^{\left(0\right)}}}$ be its complement. Note that $Q_{v^{\left(0\right)}}$ is trivially all *m*-bit strings if *l*_*w*_ < 2*h*. Let *R*_*s*_ be the set of *m*-bit strings with the lowest *h* bits all matching the bits of *s*, and let $\bar{R_{s}}$ be its complement. Let *S*_*s*_ be the set of *m*-bit strings with the *h* bits preceding index *l*_*w*_ all matching the bits of $\tilde{s}$, where $\tilde{s}$ is a cyclic permutation of *s*:

$${\tilde{s}}_{i}=s_{(i+l_{w})\;\text{mod}\;h},$$ (11)

and let $\bar{S_{s}}$ be its complement. We will make use of the fact that:

$$\begin{array}{ccc} s\neq s^{\prime } & \Rightarrow & S_{s}\cap S_{s^{\prime }}=R_{s}\cap R_{s^{\prime }}=\emptyset . \end{array}$$ (12)

Routes are constructed in 7 stages. The network topology dictates that *l*^(*t*+1)^ = *l*^(*t*)^ + 1 (mod *m*), so we let *l* = *t* (mod *m*). and that *z*^(*t*+1)^ is equal to *z*^(*t*)^ with or without the bit in index *l*^(*t*)^ inverted, depending on whether the down or down-right edge was taken at step *t*.

**Stage 1**: (0 ≤ *t* < *h*) Down or down-right edges are chosen such that the *t*th bit of *z*^(*t*+1)^ is equal to the *t*th bit of *s*. Throughout Stage 1, all nodes are within the sender’s trusted neighborhood. Throughout Stage 1, $z^{\left(t\right)}\in Q_{v^{\left(0\right)}}$. At the end of Stage 1, *z*^(*h*)^ ∈ *S*_*s*_, and *z*^(*t*)^ will remain so until the level cycles to 0 at *t* = *m*.
**Stage 2**: (*h* ≤ *t* < *l*_*w*_ − *h*) Edges are chosen to make the *t*th bit of *z*^(*t*+ 1)^ the inverse of the *t*th bit of *z*^(0)^. Note that this stage does not occur when *l*_*w*_ < 2*h*. If this stage occurs, then $z^{\left(t\right)}\in \bar{Q_{v^{\left(0\right)}}}$ until these levels are reached again in stage 6.
**Stage 3**: (*l*_*w*_ − *h* ≤ *t* < *l*_*w*_) The bits of *z*^(*t*)^ are chosen to match $\tilde{s}$, such that after the stage is complete, *z*^(*t*)^ ∈ *R*_*s*_.
**Stage 4**: (*l*_*w*_ ≤ *t* < *m*) Paths are chosen such that the *t*th bit of *z*^(*t*+1)^ matches that of the destination node *z*_*w*_. This stage will not occur if *l*_*w*_ > *m* − *h*.
**Stage 5**: (*m* ≤ *t* < *m* + *h*) There are two cases. If 2*h* < *l*_*w*_ < *m* − *h*, then there is no overlap between the indices defining *R*_*s*_ and *S*_*s*_. In this case, the first *h* bits of *z*^(*t*)^ are set to match *z*_*w*_. Otherwise there is some overlap between the indices defining *R*_*s*_ and *S*_*s*_. In this case, the each of the first *h* bits of *z*^(*t*)^ is either kept the same if *l*_*w*_ − *h* ≤ *l* < *l*_*w*_, or set to the corresponding bit of *z*_*w*_ otherwise. In this stage and after, *z*^(*t*)^ is no longer guaranteed to be in *R*_*s*_. However, *z*^(*t*)^ remains in *S*_*s*_ during and after this stage.
**Stage 6**: (*m* + *h* ≤ *t* < *m* + *l*_*w*_ − *h*) In this stage, edges are chosen to set the bits of *z*^(*t*)^ to their corresponding value in *z*_*w*_. $z^{\left(t\right)}\in \bar{Q_{v^{\left(0\right)}}}$ throughout this stage, but not afterwards.
**Stage 7**: (*m* + *l*_*w*_ − *h* ≤ *t* < *m* + *l*_*w*_) The *h* bits of *z*^(*t*)^ preceding index *l*_*w*_ are set to match *z*_*w*_. All nodes in this stage are within *h* hops of *w* and thus in its trusted neighborhood. After this stage, $v^{\left(m+l_{w}\right)}=w$ and routing is complete.

### Running time

At each node, the choice of edge is made using *O*(1) lookups of the endpoint values at index *t* and possibly *O*(1) lookups of the value at a particular index of *s*. For an *m*-dimensional butterfly network, the algorithm chooses edges at most 2*m* = *O*(*m*) times. In an *m*-dimensional butterfly, there are *N* = *m*2^*m*^ nodes; *m* = *O*(log *N*). The total running time to calculate a single path is thus *O*(*klogN*). Sending a message with redundancy *k* thus requires *O*(*k*log*N*) time.

### Proof of path independence

**Theorem 1**. *Given an m-bit wrap-around butterfly network* (*m* > 1), *and an integer h* ($1\leq h\leq \lfloor \frac{m}{2}\rfloor$), *for all node pairs* (*v*, *w*) *such that d*(*v*, *w*) ≥ 2*h, there exist at least* 2^*h*^ *h-internally vertex disjoint paths v*_*s*_ (0 ≤ *s* < 2^*h*^) *from v to w such that s* ≠ *s*′ ⇒ *v*_*s*_ ∩ *v*_*s*′_ ⊂ *T*_*h*_(*u*) ∪ *T*_*h*_(*v*).

*Proof*. Nodes from two paths can only coincide if their levels are the same. Nodes which share a level must either be in the same stage, or 4 stages apart. Let (*a*,*a*′) denote a pair of sub-paths corresponding to stage *a* of one path and stage *a*′ of another. Excluding paths that intersect in their trusted neighborhoods, (1,1) and (7,7), we have reduced the list of possible intersections to the following cases: (2,2), (3,3), (4,4), (5,5), (6,6), (1,5), (2,6), and (3,7). Nodes in stages 2–4 belong to *R*_*s*_ so cannot overlap with any stage 2–4 nodes from another path, eliminating (2,2), (3,3), and (4,4). Similarly, nodes in stages 4–6 belong to a unique *S*_*s*_, eliminating (5,5) and (6,6). Nodes in stage 1 belong to $Q_{v^{\left(0\right)}}$ while those in stage 5 belong in its complement, eliminating (1,5). Similarly, for all *l* in stage 2, *z*^(*l*)^ is equal to *z*^(0)^, while in stage 6, *z*^(*l*)^ is the inverse, eliminating (2,6) This leaves only (3,7), a collision which can occur only for only one path (with *s* matching the first *h* bits of *z*_*w*_), and which enters the trusted neighborhood in stage 3. For this single path, we can proceed directly from stage 2 to stage 7, eliminating the last possible collision.

Thus, assuming the partial trust model with trust transitive for *h* hops, we can construct 2^*h*^ paths on a wrap-around butterfly topology which do not intersect outside the trusted neighborhoods of the source and destination. Note that the node sequence $v_{s}^{\left(t\right)}$ can be calculated entirely from the source *v*, destination *w*, and path parameter *s*, meaning that with this information nodes are able to determine which neighbor to route a given message copy to. Each node in a directed wrap-around butterfly network has an out-degree of 2, placing an upper bound of 2^*h*^ on the effective redundancy, which we have just shown the above algorithm achieves, so the bound is tight. Thus, decentralized, redundant, structured networks such as the butterfly can have a very low probability of failure when faced with adversarial faults, even from a very powerful attacker.

## Discussion

Our work has been motivated by the vulnerability of current communications infrastructure to surveillance and censorship, which are often achieved by coercive targeted attacks against central nodes. We have already discussed two such cases: Pakistan’s inadvertant censorship of YouTube [12] and the FBI’s surveillance and censorship of Lavabit [13].

The reader may wonder how our methods could be employed in scenarios such as large-scale state-sponsored censorship [46]. Censorship-resistant infrastructure often replaces central servers (e.g., the router in the 2008 YouTube incident) with multiple servers across the world, synchronized through consensus protocols. The *directory authorities* used by the Tor project [47] are one example. However, the size of such networks is limited by the number of trusted relationships (degree) each node can maintain, and the inherent insecurity of extending transitive trust to an ever-larger network. Our work provides both a theoretical framework and a specific example of how network structure can be engineered to leverage trust for a high level of attack-tolerance, without sacrificing scalability.

We have focused primarily on adversarial faults that block or change messages (censorship) but our work is also relevant to surveillance. While cryptographic anti-surveillance techniques exist, they remain vulnerable to man-in-the-middle attacks, in which an intermediate node masquerades as the destination. Such attacks can be detected if the original message reaches the true destination unaltered, which SMFT can help to ensure.

In its current form, our work has several limitations. Most obviously, it requires complete control over the network structure. However, we have shown that even partially control over network structure can improve attack tolerance properties. Still a more flexible network structure is desirable. There is also the question of how to construct such a network without a central authority. This limitation may not be as severe as it seems, due to the nested structure of the butterfly network. We conjecture that smaller independently-formed networks could be merged into a single larger network without central coordination. When nodes and edges exist in geographic space (as in cables connecting internet routers) this scheme might require connections between very distant locations. Such connections are extremely expensive to construct and maintain, although many exist today in the form of redundant internet backbones and undersea cables. An alternative solution might involve satellite links, which connect distant geographic points much more easily. While turning our network theoretical results into practical applications will require considerable additional work, we believe that work is inevitably necessary in order to create attack-tolerant networks.

In addition to addressing the above limitations, we see several potential directions for future work. The development of new structured networks or multipath routing algorithms could achieve higher levels of redundancy and attack-tolerance. It is also desirable to examine how changes to social dynamics could shift self-organized networks towards a more decentralized structure. Finally, our results could be implemented to address specific applications, e.g., secure messaging, domain name resolution, or anonymous web browsing.

## Conclusion

Coercion-resistant, topological approaches to attack tolerance are needed to address the current vulnerability of communications infrastructure to censorship and surveillance. We have presented a novel concurrent multipath routing (CMR) algorithm for the butterfly network, as well as a structured multipath fault tolerance (SMFT) scheme, which can be combined to create a coercion-resistant, attack-tolerant point-to-point communication architecture. We have also shown how assuming bounded trust transitivity can enable a quantitative analysis of the relationships between network structure, trust, and attack-tolerance. In our architecture, the probability of an adversary causing an undetectable error decreases exponentially with the network’s effective redundancy. The effective redundancy, in the case of the butterfly topology, grows exponentially with the radius of trust transitivity. Furthermore, a small increase in the number of messages sent (traffic volume) can compensate for a large increase in the number of messages compromised by an adversary. These results require some control over the structure of a network, or some portion of the network. Even when network structure cannot be perfectly controlled, we have shown that partially rewiring a snapshot of the internet’s router network can greatly increase its attack-tolerance properties. We believe that this work provides a foundation for the development of additional topology-based communication architectures to guard against technical and coercive adversarial attacks, including censorship and surveillance.
